# The quest for more effective vaccine markets – Opportunities, challenges, and what has changed with the SARS-CoV-2 pandemic^☆^

**DOI:** 10.1016/j.vaccine.2022.07.032

**Published:** 2024-04-08

**Authors:** Tania Cernuschi, Stefano Malvolti, Shanelle Hall, Luc Debruyne, Hanne Bak Pedersen, Helen Rees, Emer Cooke

**Affiliations:** a Department of Immunization, Vaccines and Biologicals, World Health Organization, Geneva, Switzerland; b MMGH Consulting GmbH, Zurich, Switzerland; c The Yellow House, Seattle, WA, USA; d The Yellow House, Copenhagen, Denmark; e Access-to-Medicines Research Center, KU Leuven, Leuven, Belgium; f UNICEF Supply Division, Copenhagen, Denmark; g Wits Reproductive Health and HIV Institute, University of the Witwatersrand, Johannesburg, South Africa; h Chairperson of the South African Health Products Regulatory Authority Board, South Africa; i European Medicines Agency, Amsterdam, North Holland, Netherlands

**Keywords:** Vaccines, Vaccine supply, Vaccine access, Market preparedness, COVID-19

## Abstract

The past two decades have seen important progress in access to timely, reliable, affordable, and quality-assured supplies of vaccines of global public health importance. The new vaccines developed are powerful tools to fight killers such as pneumonia, diarrhea, and cervical cancer. Global and regional financing and pooled procurement have shortened the lag between access in high- and lower-income countries.

The COVID-19 pandemic has shown that by addressing shortcomings and seizing opportunities, we can do even more. In response to COVID-19, vaccine development and access shifted from a sequential, risk-averse paradigm to a rapid approach with maximum compression of time to market while ensuring quality. Vast public investments and innovative technologies were key facilitators. The pandemic has shown that governments play a crucial role in investing in new vaccines and manufacturing capacity and sharing risks with industry. Despite impressive progress, equity in access remains elusive with important moral, economic, and health-related consequences.

Global leaders are working on a new International Treaty for Pandemic Prevention, Preparedness, and Response. To apply the lessons of COVID-19, that treaty should include a new paradigm for access to vaccines in which governments agree to:

(1) establish early sharing of information about emerging outbreaks and **early, evidence-informed strategic goals and leadership** that serve the collective global health interest.
(2) **shoulder risks and invest aggressively** to address the needs of today and prepare for future emergencies.
(3) **strengthen market preparedness** by investing in new vaccine technologies, regional research, development and manufacturing hubs, and insurance; by enabling regulatory harmonization; by driving market transparency and oversight; and by ensuring that where public funds are invested there is a contractual obligation to ensure access.
(4) **define principles and operational details for collaboration in times of scarcity** that enable countries to protect their own citizens while ensuring that no country is left behind.

This would ensure that COVID-19 catalyzes a shift toward greater access for all under Immunization Agenda 2030.

## 1 Introduction

Achieving the Immunization Agenda 2030 (IA2030) goal of “immunization for all” requires timely, reliable, and affordable access to appropriate vaccines of assured quality for all countries, regardless of size and income. Access to vaccines and supply security require shared understanding, common goals, and coordinated efforts from governments, industry, and international entities to achieve and maintain healthy vaccine markets.

IA2030 has identified four key strategic focus areas necessary to achieve access to supply: (a) innovation and affordability; (b) improved vaccine forecasting, procurement, and supply; (c) improved sources of assured quality vaccine; and (d) strengthened mechanisms for rapid access in emergencies [1]. Now the COVID-19 pandemic has sparked more critical and innovative thinking, and the immunization community must take stock of the new lessons and look at the next decade with a fresh mindset.

This article describes the evolution of the vaccine markets in the last two decades, captures lessons from COVID-19, and highlights the opportunity for more ambitious global action to expand sustainable access to vaccines for all and to enable the success of IA2030.

## 2 The last two decades: Success and challenges

Significant progress has been achieved in access to vaccines. Various initiatives have increased awareness of market dynamics and enabled transparent monitoring of market health. These include the Gavi Alliance’s Healthy Market Framework and UNICEF’s Vaccine Security Vision [2], [3]. According to these frameworks, healthy markets must provide reliable access to sufficient quantities of quality-assured and affordable vaccines, have a sufficiently large and diverse supplier base, and allow for innovation to meet evolving needs.

Table 1 summarizes the state of key vaccine markets along some of these dimensions. While progress has been made in clinical development, manufacturing, distribution, and demand for vaccines, important challenges remain, especially in supply–demand balance and market concentration. World Health Assembly resolutions continue to call for greater access to vaccines [4].

**Table 1:** Market attributes for key vaccines.

Vaccine (study year)^a^	Breadth: total producers	Supply-demand balance^b^	Concentration: share for 2 largest producers^c^	Reach: vaccines with potential for wide distribution^d^	Innovation: pipeline vaccines in Phase III
Bacille Calmette-Guérin (BCG, 2019)[5]	22	Concerning	50%	4	1
Human papillomavirus (HPV, 2022)	4	Concerning	99 %	3	3
Pneumococcal conjugate vaccine (PCV, 2020)	5	Concerning	97 %	3	4
Pneumococcal polysaccharide (2020)	4	Concerning	66 %	1	1
Measles (2020)	7	Balanced	96 %	2	1
Measles-rubella (2020)	6	Balanced	92 %	2	2
Measles-mumps-rubella (MMR, 2020)	7	Concerning	69 %	3	1
Penta-wP (2019)	10	Balanced	50 %	6	4
Hexa-aP (2019)	3	Concerning	100 %	2	1
Tetanus-diphtheria (2019)	17	Balanced	73 %	5	1
Inactivated Polio Vaccine, stand-alone (2019)[6]	10	Balanced	70 %	4	4
Rotavirus (2020)[7]	4	Balanced	74 %	4	2

a This table includes only vaccines for which a global market assessment is currently available from the World Health Organization (WHO) or UNICEF. Source is MI4A Market Studies, unless otherwise specified.[8] Results from market studies conducted before the COVID-19 pandemic may not reflect current market conditions.

b Supply-demand balance is assessed by the MI4A Market Studies. **Balanced**: Supply exceeds demand by sufficient margins to minimize the risk of shortages. Margins required include buffer stocks and range from 30 to 50% depending on vaccine-specific market conditions. **Concerning**: Supply exceeds demand by at least 10% but margins are not sufficient to minimize the risk of shortages. **Unbalanced**: Supply exceeds demand by less than 10%, or falls short of demand, leading to a high risk of delays in vaccine introductions and interruption of existing programs.

c Producers of bulk vaccines.

d Includes prequalified vaccines and vaccines with a large number of country registrations.

### 2.1 Progress in clinical development

#### 2.1.1 Success in developing new vaccines, but many targets remain unmet

Significant progress has been achieved in development of new vaccines against diseases that primarily affect lower-income countries (Table 2) as well as in vaccines targeting richer markets.

**Table 2:** New vaccines approved since 2010.

2010 – First conjugate vaccine for meningococcal meningitis A 2012 – First vaccine for hepatitis E 2012 – First quadrivalent (4-strain) influenza vaccine 2015 – First vaccine for enterovirus 71 2015 – First vaccine for malaria 2015 – First vaccine for dengue fever 2019 – First vaccine for Ebola 2020 – First vaccine for COVID-19

Several of those vaccines were enabled by philanthropic organizations like the Bill & Melinda Gates Foundation and the Wellcome Trust; public institutions like the United States’ Biomedical Advanced Research and Development Agency (BARDA); non-governmental organizations such as PATH; industry; and financing mechanisms such as Gavi. These organizations increased likelihood of demand, provided funding and technical assistance for clinical development, and helped finance manufacturing capacity. More recently, the Coalition for Epidemic Preparedness Innovations (CEPI), working on vaccines against pathogens with epidemic potential, and the WHO Research and Development (R&D) Blueprint, a global strategy and preparedness plan that allows the rapid activation of R&D activities during epidemics, have supported the development of vaccines against emerging infectious diseases [9], [10].

Despite this progress, vaccines are still missing for neglected diseases such as hookworm, schistosomiasis, and leishmaniasis [11], and for diseases and pathogens prioritized by the R&D Blueprint, such as Zika, Lassa Fever, Nipah and henipaviral diseases, Rift Valley Fever, Crimean-Congo hemorrhagic fever, and filoviruses. The blueprint also prioritizes R&D preparedness for “Disease X”, which refers to pathogens currently unknown to cause human disease [12]. National life-science strategies play a huge role in enabling basic research and investing in public–private partnerships for clinical development, which can influence the future availability of essential health products such as vaccines.

#### 2.1.2 Improvements in product suitability, but demand remains difficult to predict

The need for clearer development targets has been demonstrated multiple times. A rotavirus vaccine lacked vaccine vial monitors and had a large cold chain footprint [13]. The first malaria vaccine required a multi-year pilot program to address questions related to its public health use, due to vaccine characteristics that were perceived as an obstacle to broader roll out [14].

In the last decade, several mechanisms have been launched to provide guidance on product suitability and value. Gavi’s Vaccine Investment Strategy (VIS), Full Public Health Value Propositions (FPHV) by WHO, and preferred product characteristics (PPCs) from CEPI and WHO have provided more visibility on target product characteristics and potential funded demand. VIS, FPHV, and PPCs help private entities assess commercial opportunities and public health need when investing in development and manufacturing.

While helpful, this guidance does not provide assurance that demand will materialize, even for products that meet targets. Size and likelihood of demand become clear only after completion of clinical development, well after key decisions on product presentations and manufacturing capacity must be made. For markets with less commercial value, this can result in a lack of investment, suboptimal products, and delays in availability. Government strategies that take effect early in the product lifecycle to better mitigate the risks of vaccine development and manufacturing scale up are needed to promote rapid availability of suitable products.

### 2.2 Progress in manufacturing

#### 2.2.1 Success in assuring vaccine quality, but not enough regulatory harmonization

Quality assurance is essential for vaccines, which are routinely administered to healthy individuals. Real or perceived quality issues can compromise trust in immunization, rapidly erasing progress [15]. Regulatory agencies, including regional agencies such as the European Medicines Agency, and regional networks such as the African Vaccine Regulatory Forum (AVAREF), are helping to strengthen regulatory capacity and improve cross-country regulatory coordination [16]. Increased use of reliance practices under AVAREF leadership has reduced complications, resulting in broader geographical reach of newly developed vaccines such as for Ebola. Through its prequalification program, WHO has been providing regulatory services for countries procuring through United Nations agencies. WHO has also supported countries to develop stable, well-functioning and integrated regulatory systems with streamlined processes and predictable timelines [17]. Nearly 100 % of the globally distributed vaccine doses used in national immunization programs are now deemed of assured quality [18].

Even so, regulatory processes remain challenging, delaying access. Regulatory experts, manufacturers, WHO and other stakeholders have highlighted the divergence in vaccine registration requirements worldwide and pointed to the need for stepping up convergence initiatives, reliance, and improving pharmacovigilance [19]. Meeting divergent requirements is more cumbersome for developing country manufacturers, potentially limiting access to the more affordable products [20], [21]. While many regional initiatives have been established to facilitate reliance between regulators, governments must accelerate progress on this front if regulatory roadblocks are to be addressed. This should be paired with investment in efficiency of national pharmaceutical regulation [19]. Additionally, global guidance should foster more collaboration with lower-income country regulators.

#### 2.2.2 Growing supplier base, but constrained manufacturing capacity

As of 2019 the vaccine supplier base was concentrated in five large suppliers. GlaxoSmithKline (GSK), Pfizer, Merck, Sanofi, and Serum Institute of India represented together approximately 90 % of global vaccine value and a large share of the total volumes [18]. While COVID-19 vaccine manufacture has changed these figures, concentration remains, due to multiple factors. Vaccine manufacturing has high costs of entry and can yield lower profits compared to other pharmaceuticals and relative to entry costs; historically vaccines have represented a mere 2 % of the overall pharmaceutical market. Risks of failure and economies of scale also contribute to supplier concentration. Lastly, potential product liability, as vaccines are administered to healthy people, can be seen as disincentive to entry [22].

The manufacturing base is growing as a result of investments in new vaccines (discussed above). In addition, national manufacturers for large countries such as Brazil, China, India, Indonesia, and Russia are looking beyond domestic markets toward opportunities for export. This has increased the global supplier base and the number of vaccines available, particularly from India and China. Finally, the expansion of the WHO prequalification program and strategic procurement by the Pan American Health Organization (PAHO) and UNICEF have also contributed to this trend [23], [24].

On a vaccine-by-vaccine basis, however, risks and constraints remain apparent, as shown in Table 1. Many vaccines have a high level of supplier risk, with only 2 or 3 suppliers with products available for use across many countries. Supplies of vaccines have increased more slowly than demand from countries, as seen in recent shortages of products such as HPV vaccines and inactivated polio vaccines [25], [26], [27]. In addition, production has often not increased quickly in response to surges in demand, as can occur with a new vaccine or immunization strategy, or due to disease outbreaks.

These supply constraints are in part due to the technologies currently used in vaccine manufacturing. Vaccines are complex biological products and underestimating the complexity of process scale up can lead to delays in availability. Vaccines are typically manufactured in product-specific facilities and product changeovers are cumbersome, limiting flexibility. Increasing capacity requires significant lead times and financial investment. Technology transfer or changes in manufacturing technologies and processes can address these limitations, but they are costly. The quality assurance and regulatory review processes required to ensure that changes do not affect the quality of the final product also contribute to complexity.

Protected by know-how and intellectual property rights, and faced with uncertain demand, relatively low prices, the risks of vaccine hesitancy, and long-term displacing competition from superior or lower-cost products, the private sector has little incentive to invest in new manufacturing technologies and processes, or in increasing manufacturing capacity. Incentives are not aligned with maximization of social welfare and this needs correction through more incentives and public intervention. Partnerships for HPV and meningitis vaccines indicate that technology transfer may succeed if conducive environments are put in place [28], [29].

### 2.3 Progress in distribution

#### 2.3.1 Faster access for lower-income countries, but access lagging for middle-income countries

Historically, development costs and capital investments have been recouped through higher-priced sales in high-income countries (HICs), and thereafter vaccines were also made available to lower-income countries at lower prices and in large volumes. Uncertainties on demand and ability to pay, combined with complex procurement practices and payment terms, led manufacturers serving lower-income countries to focus first on private markets rather than support introduction in national immunization programs. This meant there was a delay in vaccine being made available to the public sector in lower-income countries [30].

While the paradigm has not changed, there has been substantial progress over the past 20 years with the creation of Gavi, the Vaccine Alliance. Building on the pioneering work of UNICEF and the PAHO Revolving Fund to support vaccine purchases by lower-income countries, Gavi and partners have made huge progress in improving access to vaccines for lower-income countries and in technical assistance for immunization by ensuring predictable funding and streamlined processes [31], [32].

Meanwhile, middle-income countries (MICs) that are not eligible for Gavi support have struggled to introduce or sustain new vaccines. Affordability is often quoted by countries as the major barrier and has often made the headlines in public media, but the root causes for poor access are more wide-ranging. Beyond affordability, these include insufficient political will for immunization, constrained budgets, unpredictable decision making and planning, ineffective procurement and market shaping by and on behalf of MICs, non-transparent pricing arrangements, and weaknesses in immunization programs and overall health systems [33], [34]. Global and regional efforts are attempting to tackle access issues for MICs [18], [35], [36]. These include initiatives by the African Union, small island developing states, the Association of Southeast Asian Nations, Eurasian Economic Union countries, and the South Eastern European Health Network [37], [38], [39], [40]. These mechanisms can also support access for countries that due to economic growth are no longer eligible for Gavi support.

#### 2.3.2 Improvement in market transparency, but need to consistently use information

According to economic theory, greater transparency and less information asymmetry will improve vaccine market efficiency. The need for transparency was codified in a WHA resolution on market transparency [41]. Efforts by national governments, UNICEF, PAHO, Gavi, and WHO have substantially improved transparency, creating a store of publicly available data on over 90 % of the global vaccine market [8], [42]. This transparency has increased countries’ negotiating power; improved planning, budgeting, and procurement processes; and enabled a deeper understanding of global and regional market dynamics [35].

This newly available information and improved understanding of market dynamics should now be consistently leveraged to inform collective strategies to secure access. In particular, this new level of knowledge should inform disease control strategies from their inception. For example, ambitious disease control goals that are set to drive investments could be accompanied by timely and proactive use of necessary adaptations to constrained supply , such as flexible dosing arrangements or dose-sparing strategies, until market conditions are conducive to broad access [43]. Similarly, given the high degree of demand concentration in vaccine markets, information on vaccine supply limitations could allow buyers to coordinate and negotiate more effective distribution of supply, for achievement of global health goals [44].

#### 2.3.3 Demand is strong, but it needs to be more informed and coordinated

Compared to other pharmaceuticals, demand for vaccines is concentrated among a smaller number of procurers. Governments directly procure 60 % of all doses globally and much of the remainder is procured by pooled procurement mechanisms that support lower-income economies [18], [31], [45]. Additional countries, including middle- and high-income countries, are setting up pooled procurement mechanisms [46], [47], [48].

Concentrated and informed demand is a powerful tool to counter the oligopolistic nature of vaccine supply towards enhanced access. Nevertheless, market work has tended to neglect the demand side. In addition to challenges in predicting demand early enough to inform investment and risk-sharing decisions, market segmentation due to differing product characteristics and consumer preferences can compromise access. Lower- and middle-income countries need both greater ability to inform product development and manufacturing scale up for preferred products and greater access to the evidence needed to make tradeoff decisions. Vaccines deemed functionally equivalent by WHO still have distinct characteristics and may not be operationally interchangeable. As a result, markets for PCV and HPV, rotavirus, and pertussis vaccines are segmented and a preferred vaccine may be in short supply even when the total availability is sufficient [43], [49], [50], [51]. Product preferences can have negative consequences when they delay introductions and therefore require careful consideration of tradeoffs [52].

Some countries also struggle with weak planning, forecasting and procurement processes. These issues on the demand side have led to national shortages of vaccines, including vaccines that have been on market for long time, such as the BCG vaccine for tuberculosis [8], [53]. To address this need, IA2030 and Gavi’s market shaping strategy prioritize support to countries for informed, evidence-based and timely planning and decision making on vaccine products.

Finally, all countries would benefit from better leveraging global demand information and by enhancing coordination to better guide investment and use of often scarce supply.

## 3 What has changed with COVID-19

The vaccine ecosystem has been profoundly transformed by the COVID-19 pandemic. In response to the enormous political pressure to reduce the human and economic impact of the pandemic, and to the staggering costs of disease and economic stimulus packages, vaccines have been developed and billions of doses deployed in the shortest time possible [54].

Pharmaceutical companies, biotech firms, academic institutions, military research units, and state-owned actors redirected their efforts to identifying suitable technologies and moving vaccine candidates quickly through clinical development, registration, commercialization, and distribution [55], [56]. Within 11 months from the sharing of the SARS-CoV-2 genome sequence, the first vaccines received WHO Emergency Use Listing and full marketing authorization in the first countries [56], [57]. A process that takes an average of 10 years and has never taken less than 4 years was compressed to the bare minimum. As of March 2022, 35 vaccines have been approved for use [56]. Vaccine developers in China and Russia have brought their own new vaccines to market almost concurrently with the multinationals. These countries, together with India, have rapidly scaled up production, including through technology transfer, licensing, and contract manufacturing agreements.

### 3.1 Shift from sequential to parallel processes with accelerated timelines

To achieve these successes, the previous pattern of sequential vaccine development, manufacturing, and commercialization was abandoned. Large public investments and joint planning of clinical development, regulation, and manufacturing capacity enabled working in parallel in many parts of clinical development and commercialization. This entailed a much higher level of financial risk, for example in starting large and expensive Phase III clinical trials and in building commercial-scale manufacturing facilities before achieving high confidence on the likelihood of success [58], [59]. Very importantly, safety has not been compromised and COVID-19 vaccines have been submitted for approval with larger efficacy datasets than is typically seen for other vaccines. Furthermore, extensive post-approval data are being collected in diverse target populations to expand the datasets for safety and effectiveness.

In addition, pragmatic requirements for efficacy, presentation, cold-chain requirements, duration of protection, and shelf life have been stipulated for new vaccines. These products have been launched with the expectation that continued development and subsequent products will address early product limitations [60], [61].

Early, continuous, and transparent engagement between regulators and vaccine developers and collaboration among regulators have led to addressing emerging questions and issues more efficiently. This has increased the success rate of development activities. The International Coalition of Medicines Regulatory Authorities and WHO have achieved exceptional regulatory convergence, and the European Medicines Agency has opened its procedures for COVID-19 vaccines to allow expert involvement from WHO and from international regulators [62]. WHO has involved regulatory agencies of reference in review of manufacturing dossiers for the prequalification process, providing safety and quality assurance for countries anywhere in the world, particularly lower- and middle-income countries. Through the COVAX Facility, countries had an opportunity to opt to use COVID vaccines prior to WHO Emergency Use Listing, by relying on emergency use authorization from WHO-listed regulatory authorities [63]. Clinical trials were undertaken in many lower- and middle-income countries in different regions [64]. Technical support and international collaboration in recent years set the stage for many of these actions [65]. Due to these measures, regulatory requirements have not been seen as the main bottleneck for access to COVID-19 vaccines [66].

Production of COVID-19 vaccines has also been scaled-up with unprecedented speed. This has been enabled by large public contracts to build manufacturing capacity and by advance purchase agreements where countries or pooled procurement entities guarantee purchase of a certain number of doses should a vaccine obtain regulatory approval. Without such incentives, companies would have limited interest in investing in large manufacturing plants to be potentially dismantled in a short time frame, particularly in face of relatively low pricing in high income settings [67].

### 3.2 New technologies promising rapid, flexible, and scalable response

New vaccine platforms have also made an important difference with COVID-19. These new platforms enable vaccines to be produced by incorporating genes for different proteins with little or no alteration in manufacturing process or components. Both Moderna and Pfizer/BioNTech were working on mRNA vaccines before the pandemic and could replace the mRNA they were working on with sequences from SARS-CoV-2. Work on viral-vectored platforms initiated with the Ebola vaccine could also be quickly leveraged. The proof of concept provided by the COVID-19 pandemic “for pre-emptive and reactive vaccine design, as well as faster development and manufacturing options, will permanently change our ability to rapidly respond to emerging viruses” [68]. Vaccine platforms give significant advantages along four dimensions:

(a) **Faster clinical development** – vaccine platforms, and in particular nucleic acid and viral vector technologies, allow vaccine development based on sequence information alone. If the viral antigen is known, the availability of coding sequences for this protein suffices to start vaccine development. As a result, these platforms are highly adaptable and speed up vaccine development considerably [68].
(b) **Streamlined regulatory requirements** - because they can be used for multiple targets, vaccine platforms can be considered as candidates for unique approaches to regulation across multiple vaccines [69]. Platforms share a common backbone and only the antigenic target is varied, hence the platform itself might be accepted by regulatory agencies. Evaluation would still be needed for the new target, given that immunogenicity and autoimmune considerations are antigen-specific. Streamlined regulatory requirements have been widely used for influenza vaccines and were adopted by the WHO for Ebola vaccines.
(c) **Agile manufacturing** - a platform-based approach can provide a flexible base for vaccine manufacturing that can be more easily repurposed for use with new products. This flexible base could even enable multiple candidates to be developed in parallel using the same platform. Additionally, these platforms could enable increased use of contract manufacturing organizations and allow vaccine variants to be generated and evaluated potentially without requiring the manufacturer to modify or revalidate production processes [69]. This represents a paradigm shift for an industry that usually dedicates one building or production area to each vaccine.
(d) **Investment de-risking** - because vaccine platforms can be leveraged for multiple uses across the continuum of clinical development and manufacturing, they enable economies of scale and flexibility. This lowers the risk of vaccine ventures, in particular for pandemic response and emerging infectious diseases.

### 3.3 Greater public intervention, but even more is needed

As reviewed above, unprecedented, large, and fast public investment by governments has been a key enabler to great successes in developing and manufacturing COVID-19 vaccines complementing private investment. For instance, public research accounted for 70 % of clinical trials for COVID-19 vaccines [70]. We should applaud these efforts which led manufacturers to commit to over 10 billion doses of COVID-19 vaccine by the end of 2021, an extraordinary tripling of global vaccine market size within a matter of months [71].

Despite these tremendous efforts, even more could have been done to meet larger global public health needs and support ambitious timelines for vaccine deployment set by countries. Throughout 2021, heads of the United Nations, UNICEF, International Monetary Fund, World Bank Group, World Health Organization, and World Trade Organization and many others repeatedly called for additional increases in manufacturing [72], [73], [74]. Further public intervention, such as larger contracts with more incentives for earlier deliveries, were needed. These contracts would have been justified based on their potential impact and the costs of inaction [75]. They were also justified in relation to risks that the COVID-19 vaccine production has posed to supply of other antigens [76].

In addition to increasing their investments, governments should also have played a more active role in oversight of vaccine markets as called upon by many [77], [78]. Plans for production of COVID-19 vaccines, allocation amounts, and delivery dates have been unclear. Lacking reliable data in this crisis, governments and other stakeholders have been struggling, especially in the first 12 months of vaccine availability, to make informed, rapid, and strategic decisions for effective distribution of vaccines. To strike a careful balance between rewarding private investments and fair and predictable access for purchasers, governments must request more transparency in manufacturing costs, capacity, contracts, and operations; they should also ensure licensing and technology transfer to enable increased production.

Unprecedented use of partnerships has enhanced manufacturing bulk production, access to proprietary adjuvants, and final product fill and finish [79], [80], [81], [82]. In addition, a network of contract manufacturing organizations and vaccine manufacturers provided capacity to scale up manufacturing under good manufacturing practices very early in the game, accelerating the transition from clinical development to commercialization, especially for smaller companies. That said, the world has judged these efforts to be insufficient, and difficult World Trade Organization negotiations over the long-sought intellectual property waiver for COVID-19 vaccines [83] point to the need for further dialogue on ways to ensure faster and more equitable access.

For future pandemics, governments could also partner with the private sector to establish additional dedicated and permanent manufacturing capacity as insurance against future needs [84]. This could be done through shared facilities at the regional or continental level with rights for governments to take over at times of pandemic. The work on the regional mRNA manufacturing hubs is a first example of this approach being implemented [85]. These facilities would need to be staffed adequately and maintained to current global standards to provide flexible manufacturing capacity. Between emergencies they could be used to manufacture vaccines for research, for stockpiling, or to serve lower-income countries while dedicated capacity is being established to serve them sustainably. The new platform technologies discussed above promise to decrease the cost of such a setup. These regional facilities would also enhance diversity in manufacturing, similarly to what has been done through BARDA and has been explored through the European Union’s Health Emergency Preparedness and Response Authority Incubator [86], [87]. Countries in Asia, Africa and Latin America have long aspired to create their own vaccine industries [88]. If know-how, intellectual property, and sustainability challenges can be overcome, these new regional mechanisms could also be leveraged to enhance routine regional vaccine provision, and improve access and affordability for all countries.

### 3.4 Leadership and collaboration are needed to address inequitable distribution

Over 180 countries have signed on to the COVAX Facility and countries with constrained resources have been served by this pooling mechanism at high speed [89]. Nevertheless, the mechanism has struggled to fulfill its aims [90]. Importantly, in spite of its efforts to coordinate global demand and supply for equitable distribution [91], most contracted volumes have sat outside of the Facility. Other pooled procurement mechanisms such the EU Vaccines Strategy and the African Vaccines Acquisition Trust [92], [93] and bilateral agreements between governments and manufacturers have rapidly secured doses, particularly for high-income countries. The richest countries have established deals for up to sixfold the doses they need to meet their goals on the assumption that a significant portion of their portfolio would fail or materialize too slowly [56].

National and regional entities have clear and strong incentives to serve their populations first through faster and more flexible contracts given high caseloads, severe disease, and deaths. Nevertheless, lack of coordination across all the existing procurement channels is short-sighted. In 2021, vaccination in lower-income countries lagged significantly behind richer countries due to lack of access. In 2022, the problem has been compounded by limited country system capacity to deploy vaccines in short timeframes [94], [95]. Delayed vaccination has not only been a moral failure, but has also meant that high risk populations in disadvantaged settings have gone without protection, with important health consequences [54]. The risk this poses to local and global economies has also been extensively discussed, as well as the potential impact on virus variants [96], [97].

National interests have also led some countries to block exports of domestically manufactured COVID-19 vaccines or raw materials when their own contracted supply has not been delivered or because of domestic political pressure [98], [99]. This has had major impact in further slowing down access – in particular to lower-income countries.

The COVID-19 experience suggests that serving all populations more equitably and ending future pandemics more rapidly cannot be achieved just through financing and procurement mechanisms: it will require a radically new mindset. We need a clear vision on the goals and objectives of a global vaccination strategy to inform bolder, coordinated investment by both public and private entities. We also need to strike a much better balance between serving national interest and global public health objectives. The only means to achieve this is through high-level diplomacy between countries, and commitment to a new paradigm.

## 4 Conclusion

Over the past two decades we have achieved important progress in access to timely, reliable, affordable, and quality-assured supply of vaccines. In the last two years, COVID-19 has highlighted the importance of vaccines for global security and the incredible cost of being caught unprepared. The pandemic has also shown us that by addressing shortcomings and seizing new opportunities, we can do more.

The question now is whether we will consider the COVID-19 experience an outlier due to a unique combination of factors, or whether we will apply its lessons to a shift toward greater access for all under IA2030.

While the lessons of COVID-19 are fresh, we have an opportunity to establish a new paradigm for vaccine development and access that builds on these new practices. This new paradigm should be incorporated as part of the International Treaty on Pandemic Prevention, Preparedness and Response. The 194 World Health Organization member states agreed in December 2021 to begin negotiations on such treaty, aiming for a draft agreement to be considered by the 77th World Health Assembly in May 2024 [100]. In this new paradigm, governments should commit to:

(1) establish early sharing of information about emerging outbreaks and **early, evidence-informed strategic goals and leadership** that serve the collective global health interest.
(2) **shoulder risks and invest aggressively** to address the needs of today and prepare for future emergencies.
(3) **strengthen market preparedness** by investing in new vaccine technologies, regional R&D and manufacturing hubs, and insurance; by enabling regulatory harmonization; by driving market transparency and oversight; and by ensuring that where public funds are invested there is a contractual obligation to ensure access.
(4) **define principles and operational details for collaboration in times of scarcity** that enable countries to protect their own citizens while ensuring that no country is left behind.

In the next pandemic, this paradigm would serve both equity and self-interest by reducing disease everywhere through a faster, more coordinated, and more equitable global response. Between pandemics, this paradigm would enable bolder, coordinated leadership to improve access to vaccines for all.

## Disclaimer

The views expressed in this article are the personal views of the authors and may not be understood or quoted as being made on behalf of or reflecting the position of the regulatory agency/agencies or organizations with which the author(s) is/are employed/affiliated.

## Data statement

There are no data associated with this manuscript.

## CRediT authorship contribution statement

**Tania Cernuschi:** Conceptualization, Investigation, Writing – original draft, Writing – review & editing, Supervision, Funding acquisition. **Stefano Malvolti:** Conceptualization, Investigation, Writing – original draft, Writing – review & editing. **Shanelle Hall:** Conceptualization, Investigation, Writing – original draft, Writing – review & editing. **Luc Debruyne:** Investigation, Writing – review & editing. **Hanne Bak Pedersen:** Investigation, Writing – review & editing. **Helen Rees:** Investigation, Writing – review & editing. **Emer Cooke:** Investigation, Writing – review & editing.

## Declaration of Competing Interest

The authors declare the following financial interests/personal relationships which may be considered as potential competing interests: LD was formerly President of Global Vaccines for GlaxoSmithKline and holds shares in the company. He is a member of the Life Sciences Advisory Board for GreenLight Biosciences Inc., and a strategic advisor to the Coalition for Epidemic Preparedness Innovations. The remaining authors declare that they have no known competing financial interests or personal relationships that could have appeared to influence the work reported in this paper.
